# A four‐long noncoding RNA signature predicts survival of hepatocellular carcinoma patients

**DOI:** 10.1002/jcla.23377

**Published:** 2020-05-30

**Authors:** Haitao Jiang, Lianhe Zhao, Yunjie Chen, Liang Sun

**Affiliations:** ^1^ Department of General Surgery HwaMei Hospital University of Chinese Academy of Sciences Ningbo China; ^2^ Ningbo Institute of Life and Health Industry University of Chinese Academy of Sciences Ningbo China; ^3^ Key Laboratory of Diagnosis and Treatment of Digestive System Tumors of Zhejiang Province Ningbo China; ^4^ Key Laboratory of Intelligent Information Processing Advanced Computer Research Center Institute of Computing Technology Chinese Academy of Sciences Beijing China

**Keywords:** biomarker, hepatocellular carcinoma, lncRNA, prognostic, signature

## Abstract

**Background:**

Hepatocellular carcinoma (HCC) is a common neoplasm located in the liver. Accumulating evidence has highlighted that long noncoding RNAs (lncRNAs) are correlated with the survival of HCC patients. This study focuses on finding a lncRNA signature to predict the prognostic risk of HCC patients.

**Methods:**

Statistical and machine learning analyses were conducted to analyze the lncRNA expression data and corresponding clinical data of 180 HCC patients collected from the public online Tanric and The Cancer Genome Atlas (TCGA) databases.

**Results:**

From the training dataset, we obtained the four‐lncRNA model comprising RP11‐495K9.6, RP11‐96O20.2, RP11‐359K18.3, and LINC00556 which can divide HCC patients into two different groups with significantly different prognosis (n = 90, median 1.81, 95% confidence interval [CI]: 1.50‐4.91 vs 8.56 years, 95% CI: 6.96‐9.97, log‐rank test *P* < .001). The test dataset confirmed the prognostic ability of the signature (n = 90, median 1.95, 95% CI: 1.14‐4.08 vs 5.80 years, 95% CI: 3.11‐6.82, log‐rank test *P* = .007). Receiver operating characteristic curve displayed the better prediction efficiency of the four‐lncRNA signature than the tumor/node/metastasis stage. Cox analysis showed the four‐lncRNA signature was an independent predictor of HCC prognosis.

**Conclusion:**

The four‐lncRNA signature can be used as an independent biomarker for HCC patients to predict the prognostic risk.

**Abbreviations**
CI: confidence interval
HCC: hepatocellular carcinoma
lncRNA: long noncoding RNA
TCGA: The Cancer Genome Atlas

## 1 INTRODUCTION

Hepatocellular carcinoma (HCC) is a refractory tumor that kills 746 000 people every year,^1^, ^2^ ranked as the third cause of cancer‐induced death. The main reasons for the high mortality of HCC are the following two points. First, the disease is insidious and difficult to be detected early; thus, most of the HCC patients are diagnosed at advanced stages when they are in poor physical condition and miss the opportunity of surgery; second, there are few effective treatments for patients with advanced HCC who are not only insensitive to radiotherapy but also poorly responsive to conventional chemotherapy drugs.^3^ In recent years, it has been recognized that molecular characteristics are closely related to the prognosis and therapeutic effectiveness of HCC patients.^4^ Therefore, identifying molecular indicators will result in more accurate prognostic judgments and improved treatments, which are urgently needed for HCC patients.

Long noncoding RNAs (lncRNAs) are a group of noncoding RNAs with the length more than 200 bp.^5^, ^6^ Recent studies have found that lncRNAs play important roles in the regulation of important biological processes in various types of cancer, especially the oncogenic or onco‐suppressive role,^7^, ^8^ implying the potential of lncRNAs as biomarkers and therapeutic targets for cancer.^9^, ^10^ In addition, the prognostic role of lncRNA in HCC has been reported in many studies. For instance, lncRNA PTTG3P was found to be associated with short survival in HCC patients and could be used as an unfavorable prognostic predictor.^11^ LncRNA ASB16‐AS1 was demonstrated to promote the malignant behavior of HCC through regulating miR‐1827/FZD4/Wnt/β‐catenin pathway and has the prognostic value.^12^ CTC‐297N7.9 was observed to be high expressed in HCC patients with good prognosis, indicating its protective role.^13^ Subsequently, due to better prediction performance than a single lncRNA molecule, lncRNA signatures for HCC prognosis prediction are being discovered.^14^, ^15^, ^16^

In the present study, we aimed to identify lncRNAs that could predict outcomes of HCC patients and construct a prognostic lncRNA signature based on lncRNA expression profile data of HCC from the The Cancer Genome Atlas (TCGA) and Tanric databases.

## 2 MATERIALS AND METHODS

### 2.1 Construction process of the lncRNA risk score model

LncRNA transcriptome expression data of 180 HCC patients were downloaded from the Tanric database (https://www.tanric.org/home).^17^ Corresponding clinical information of 180 HCC patients was downloaded from TCGA database (https://xenabrowser.net/datapages/). We omitted lncRNAs expressing value with coefficient of variance >0.1 and selected survival‐related lncRNAs from training samples by performing Cox analysis (*P* < .05). Then, we used the random survival forests‐variable hunting algorithm to further filter nodes until nine lncRNAs were screened out.^18^ We developed risk score models to estimate prognosis risk as follows ^16^, ^19^: $\text{Risk}\;\text{score}=\sum_{i=1}^{N}(\text{lncRNAexp}*\text{coefficientCOXi)}$ , where N represents the lncRNAs number in the model, lncRNAexp is the lncRNAs expression value, and coefficientCOXi is the coefficient of lncRNAs in the Cox analysis. We selected signatures which predicted the HCC OS with AUC > 0.7 and log‐rank *P* < .05 from all 2^9^‐1 = 511 signatures.

### 2.2 Statistical analysis

We used R program, including pROC, TimeROC, Survival, and RandomForestSRC (from Bioconductor: http://www.bioconductor.org/) to perform statistics and machine learning analysis. Using the receiver operating characteristic (ROC) and the Time ROC analysis,^20^, ^21^ we compared the prognostic performance of tumor/node/metastasis (TNM) stage and the lncRNA signature. Cox analysis was performed on the data processing to identify the prognostic factors with significance defined as *P* < .05. Pearson's test with *P* < .05 and the Pearson coefficient >0.2 <−0.2 were used to select co‐expressed protein‐coding genes with lncRNAs which were visualized by Cytoscape (3.2.3).^22^ We performed Kyoto Encyclopedia of Genes and Genomes (KEGG) and Gene Ontology (GO) enrichment analysis by the R package clusterProfiler.^23^

## 3 RESULTS

### 3.1 Constructing the lncRNA signature for predicting HCC prognosis in the training group

Table 1 displayed the detailed clinical information of the 180 HCC patients. The median age of the enrolled patients was 63 years (20‐90 years) including 67 female and 113 male patients. A total of 165 HCC patients were categorized as TNM stage I to IV. These 180 HCC patients were randomly divided into two groups, one as the training (n = 90) group and one as the test group (n = 90). We constructed prognostic lncRNA signature from the training group and then verified its predictive power in the test group.

**TABLE 1:** Clinicopathological parameters of hepatocellular carcinoma patients in each cohort

Characteristic	Training set	Testing set
Age (y)		
>63	48	44
≤63	42	46
Sex		
Female	28	39
Male	62	51
Vital status		
Living	59	47
Dead	31	43
Tumor/node/metastasis stage		
I	37	34
II	22	22
III	26	21
IV	1	2
Unknown	4	11

**FIGURE 1:**
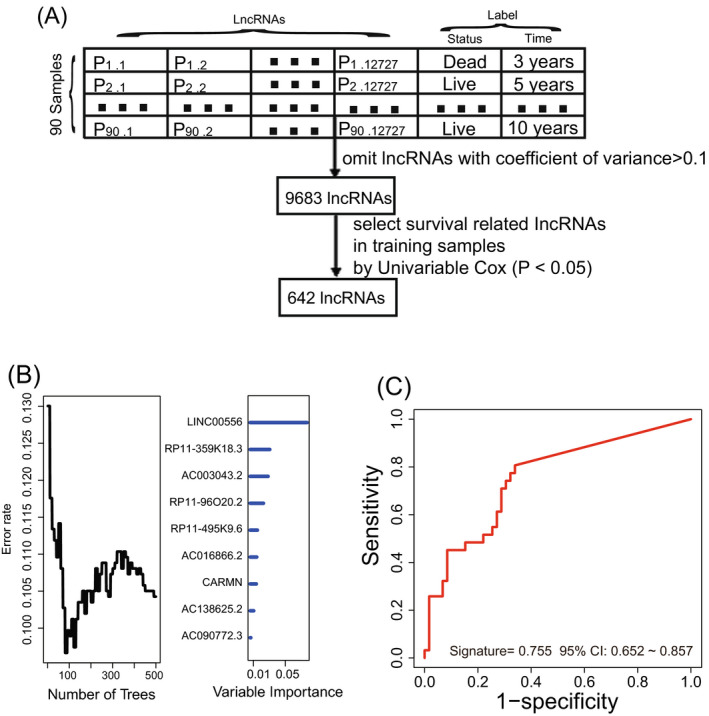
Constructing the prognostic long noncoding RNA (lncRNA) signature in the training dataset. A, The process of selecting the survival‐related lncRNAs. B, Based on the associated expression score, random survival forests‐variable hunting analysis was performed to filter lncRNAs. C, Receiver operating characteristic analysis of the selected signature

First, we selected 9683 lncRNAs with coefficient of variance <0.1 based on their expression value from 12 727 lncRNAs. Then, we used univariate Cox regression analysis and got a 642‐lncRNA set associated with HCC patient OS (Figure 1A, *P* < .05). Finally, through random survival forests analysis, we obtained 9 prognostic lncRNAs according to importance score (Figure 1A,B).

Kaplan‐Meier and ROC analyses were performed on 2^9^‐1 = 511 signatures. The lncRNA combination including RP11‐495K9.6, RP11‐96O20.2, RP11‐359K18.3, and LINC00556 was considered as the final lncRNA signature since its AUC value was the largest (AUC > 0.70) and log‐rank *P* < .001 (Figure 1C). The lncRNA signature risk score (Table 2) = (1.13 × RP11‐495K9.6 expression value) + (1.35 × RP11‐96O20.2 expression value) + (1.42 × RP11‐359K18.3 expression value) + (2.17 × LINC00556 expression value).

**TABLE 2:** The feature of the long noncoding RNAs (lncRNAs) in the prognostic expression signature

lncRNA name	Ensembl ID	Coefficient^a^	*P* value^a^	Gene expression level association with poor prognosis
RP11‐495K9.6	ENSG00000249926	1.13	.01	High
RP11‐96O20.2	ENSG00000259681	1.35	.01	High
RP11‐359K18.3	ENSG00000259788	1.42	<.001	High
LINC00556	ENSG00000260131	2.17	<.001	High

^a^ Derived from the univariable Cox analysis in the training set.

### 3.2 The predictive performance of the four‐lncRNA signature

**FIGURE 2:**
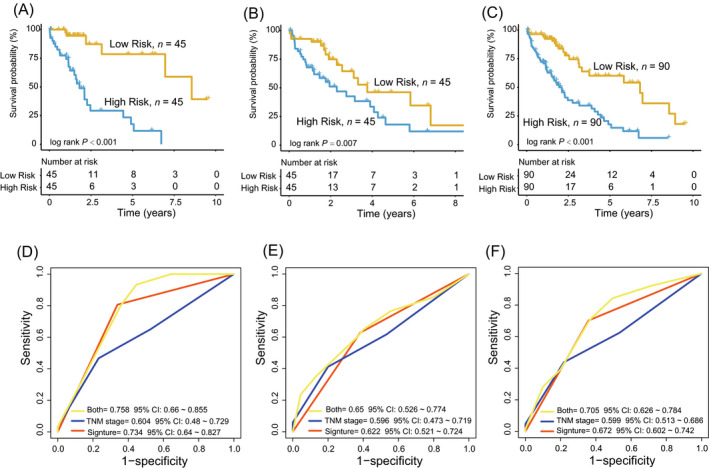
The performance of the four‐long noncoding RNAs (lncRNA) signature in Hepatocellular carcinoma prognosis prediction. A‐C, Kaplan‐Meier analysis of the SIGNATURE in the training, test, and entire The Cancer Genome Atlas datasets. D‐F, Comparing the survival prediction power between the lncRNA signature and tumor/node/metastasis stage by receiver operating characteristic in the training, test, and entire datasets

Based on the four‐lncRNA signature, HCC patients obtained their risk scores. We used the median risk score as a cutoff point for Kaplan‐Meier analysis, and HCC patients in the training group (n = 90) were subgrouped into two risk groups with significantly different survival. The median survival of the high‐risk group was shorter than that of the low‐risk group (median survival time: 1.81 years, 95% confidence interval [CI]: 1.50‐4.91 vs 8.56, 95% CI: 6.96‐9.97, log‐rank test *P* < .001; Figure 2A). Then, we test the survival predictive performance of the signature in the test set. Kaplan‐Meier result revealed the outcome of high‐risk patients were significantly different from low‐risk patients (median survival time: 1.95, 95% CI: 1.14‐4.08 vs 5.80 years, 95% CI: 3.11‐6.82, *P* = .007; Figure 2B). At last, we tested the risk identification ability of the signature in the entire TCGA dataset (n = 180) and the Kaplan‐Meier result showed that the HCC patients of the low‐risk group (n = 90) outlived ones in high‐risk group (n = 90) in Figure 2C (log‐rank *P* < .001).

### 3.3 Prognostic independence test of the four‐lncRNA signature

Chi‐square test found there was no correlation between the signature and other clinical features (Table 3). We further performed univariable and multivariable Cox analysis to evaluate the prognostic independence of the four‐lncRNA signature. As shown in Table 4, the four‐lncRNA signature was proved to be an independent indicator in the training group (high‐risk vs low‐risk, HR = 3.95, 95% CI 3.65‐8.90, *P* < .001, n = 90). The test group and the entire TCGA set verified the accuracy of the independence test (HR = 2. 38, 95% CI 1.14‐4.96, *P* = .02, n = 90; HR = 3.82, 95% CI 2.17‐6.71, *P* < .001, n = 180).

**TABLE 3:** Association of the long noncoding RNA signature with clinicopathological characteristics in the hepatocellular carcinoma patients

Variables	Train group		*P*	Test group		*P*	Entire group		*P*
	Low risk^a^	High risk^a^		Low risk^a^	High risk^a^		Low risk^a^	High risk^a^	
Age (y)									
>63	17	25	.14	21	23	.83	38	48	.18
≤63	28	20		24	22		52	42	
Sex									
Female	10	18	.11	23	16	.20	33	34	.35
Male	35	27		22	29		57	56	
M stage									
M0	39	32	.16	31	29	.27	70	61	.21
M1	0	1		2	0		2	1	
N stage									
N0	28	31	.37	29	23	.31	57	54	.62
N1	2	0		0	1		2	1	
N2	14	14		16	21		30	35	
T stage									
T1	22	17	.09	14	22	.22	36	39	.17
T2	14	9		17	9		31	18	
T3	8	17		9	10		17	27	
T4	0	2		5	3		5	5	
Tumor/node/metastasis stage									
I	20	17	.22	14	20	.20	34	37	.29
II	13	9		14	8		27	17	
III	9	17		11	10		20	27	
IV	0	1		2	0		2	1	

^a^ Low risk ≤ median of risk score; high risk > median of risk score; the chi‐squared test; *P* value < .05 was considered significant.

**TABLE 4:** Univariable and multivariable Cox regression analysis of the lncRNA signature with survival of hepatocellular carcinoma patients in the training group, test group, and entire group

Variables	The training set (n = 90)				The Test set (n = 90)				The TCGA dataset (n = 180)			
	HR	95% CI of HR		*P*	HR	95% CI of HR		*P*	HR	95% CI of HR		*P*
		Lower	Upper			Lower	Upper			Lower	Upper	
Univariable analysis												
Age												
>63 vs ≤63	0.76	0.37	1.55	.44	1.51	0.79	2.88	.22	1.09	0.68	1.74	.73
Sex												
Male vs female	1.60	0.73	3.50	.24	1.15	0.62	2.13	.65	1.26	0.78	2.03	.34
TNM stage												
IV + III vs I + II	1.36	0.90	2.06	.15	1.24	0.85	1.81	.27	1.30	0.98	1.71	.07
lncRNA signature												
High risk vs low risk	3.34	3.23	7.03	<.001	2.03	1.08	3.84	.03	3.56	2.11	6.00	<.001
Multivariable analysis												
Age												
>63 vs ≤63	0.93	0.43	2.01	.85	1.45	0.71	2.97	.31	1.18	0.71	1.98	.52
Sex												
Male vs female	2.59	1.09	6.15	.03	1.13	0.55	2.32	.73	1.34	0.80	2.22	.27
TNM stage												
IV + III vs I + II	1.10	0.71	1.70	.68	1.40	0.94	2.08	.10	1.35	1.02	1.78	.04
lncRNA signature												
High risk vs low risk	3.95	3.65	8.90	<.001	2.38	1.14	4.96	.02	3.82	2.17	6.71	<.001

Abbreviation: TNM, tumor/node/metastasis.

### 3.4 Comparison of the lncRNA signature with TNM stage system

Receiver operating characteristic analyses found that the AUC value of the lncRNA signature was greater than that of the TNM stage system in the training, test, and entire datasets (n = 90/90/180), (lncRNA model‐AUC = 0.73/0.62/0.67 vs TNM‐AUC = 0.60/0.60/0.60, Figure 2D‐F), demonstrating the lncRNA signature had better survival predictive performance. Combining the lncRNA signature and the TNM stage had the largest AUC value, indicating the signature could be used as an auxiliary prognostic marker (Both‐AUC = 0.76/0.65/0.71, Figure 2D‐F).

**FIGURE 3:**
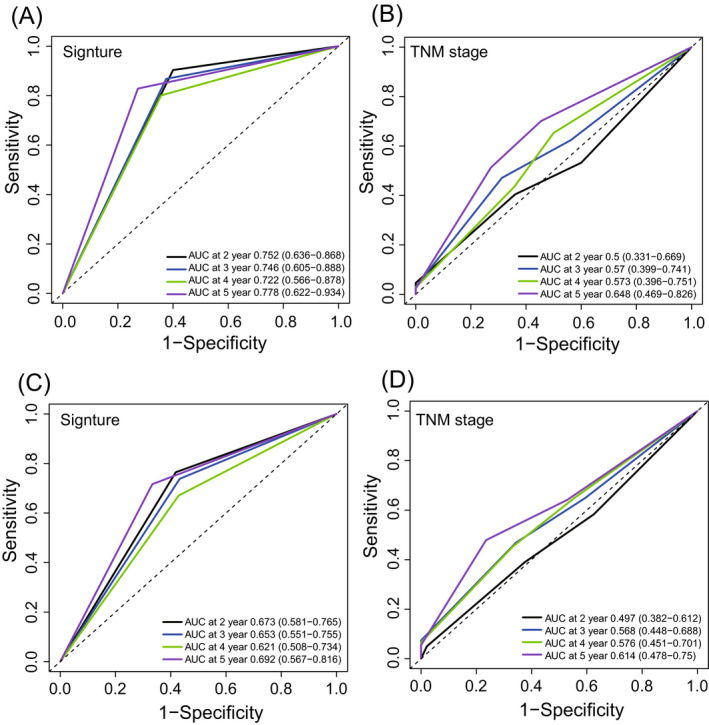
TimeROC analysis of the signature and tumor/node/metastasis stage for the survival prediction at 2, 3, 4, and 5 y in the training (A, B) and test dataset (C, D)

On the other hand, the result of TimeROC demonstrated that the predictive ability of lncRNA signature outperformed that of the TNM stage. The AUCs of the four‐lncRNA signature in the training group were 0.75/0.75/0.72/0.78 at 2/3/4/5 years, greater than the corresponding AUC values of TNM stage (Figure 3A,B). Similar results were also visible in the entire TCGA dataset (signature‐AUC training = 0.67/0.65/0.62/0.69 at 2/3/4/5 years vs TNM‐AUC training = 0.50/0.57/0.58/0.61 at 2/3/4/5 years, Figure 3C,D).

### 3.5 Stratified analysis for TNM stage

**FIGURE 4:**
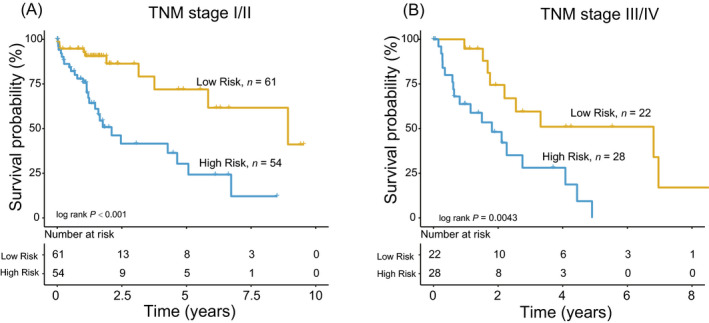
The four‐long noncoding RNA signature stratified tumor/node/metastasis low/high stage into two groups with different survival in the entire dataset (A, B)

Combined the TNM stage with lncRNA signature risk scores, we stratified the HCC patients into different subgroups. HCC patients with TNM I + II stage were stratified into high‐risk and low‐risk subgroups. Kaplan‐Meier analysis showed there was a significant difference in survival time between the two subgroups (log‐rank test *P* < .001, Figure 4A). HCC patients with TNM III + IV stage were also divided into two risk subgroups with different survival (log‐rank test *P* = .0043, Figure 4B).

### 3.6 Function prediction of the four lncRNAs in the signature

**FIGURE 5:**
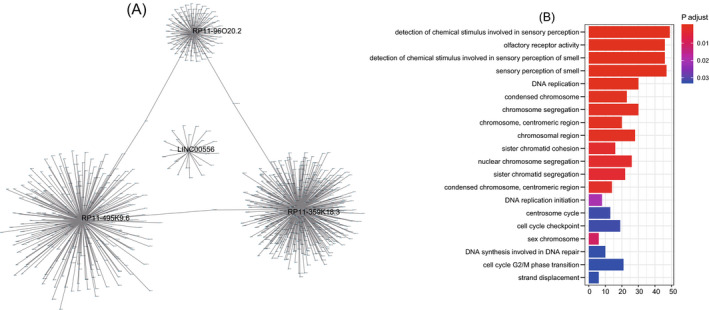
Function prediction of the four long noncoding RNAs (lncRNAs). A, The co‐expression network. B, Function analysis of the four prognostic lncRNAs

First, we used Pearson's test to compute the co‐expressed mRNAs with the four lncRNAs in the entire TCGA dataset (n = 180). A total of 749 mRNAs were selected which were co‐expressed with at least one of the four lncRNAs (coefficient >0.2/<−0.2, *P* < .05, Table S1, Figure 5A). Then, we used those co‐expressed genes to predict the biological function of the four lncRNAs. We found the four lncRNAs were enriched in 27 GO terms and KEGG pathways and the top 20 pathways were visualized in Figure 5B, such as DNA replication and cell cycle checkpoint (*P* < .05 Figure 5B).

## 4 DISCUSSION

A vast amount of research suggests that lncRNAs might serve as biomarkers in the diagnosis and prognosis of various tumors, including HCC. In addition, lncRNA has the advantage of being a marker because it is easy to detect in body fluids.^24^ Thus, there have been many articles on the prognostic lncRNA markers of HCC. Based on high throughput sequencing data, lncRNAs associated with the HCC prognosis have been identified, such as ASB16‐AS1, LINC01138, and CTC‐297N7.9.^12^, ^13^, ^25^ These lncRNAs were found play important roles in HCC carcinogenesis through regulating tumor proliferation and migration. Because of its better predictive efficacy, lncRNA signatures have been developed for prognostic prediction in many cancers such as esophageal squamous cell carcinoma, glioblastoma, lung adenocarcinoma, and pancreatic ductal adenocarcinoma, among others.^19^, ^26^, ^27^, ^28^

In this study, we collected and downloaded the expression data and clinical information of HCC cohort from Tanric and TCGA. Using statistical and machine learning analysis, we found 642 lncRNAs significantly correlated with overall survival and constructed a four‐lncRNA signature which was proved to be a reliable indicator of HCC survival in 180 samples. The independence test detected the survival prediction ability of the four‐lncRNA signature in HCC was not affected by age, gender, and TNM stage. In addition, stratification analysis discovered the four‐lncRNA signature or the four‐lncRNA– based risk score model can further subdivide HCC patients at same TNM stage into different risk groups with significantly different outcomes, suggesting that the four‐lncRNA signature can be used as an assistant prognostic model for TNM stage in HCC. Moreover, we found high expression of RP11‐495K9.6, RP11‐96O20.2, RP11‐359K18.3, and LINC00556 was correlated with poor prognosis of HCC patients (HR > 1, *P* < .05). Since the function of these four lncRNAs has not been reported yet, we performed Go and KEGG analysis and found that the coding genes co‐expressed with the four lncRNAs were enriched in terms related to DNA replication and repair, indicating that the four lncRNAs in the signature may participate in the HCC progression through DNA replication and repair related pathways. The specific mechanism of these lncRNAs regulates the prognosis of HCC remains to be elucidated.

In summary, using statistical and machine learning analyses, we constructed a four‐lncRNA signature including RP11‐495K9.6, RP11‐96O20.2, RP11‐359K18.3, and LINC00556 which could be used effectively to predict clinical outcome of HCC patients. The four‐lncRNA signature exerts great applicable value in prognosis prediction, therapy selection, and disease recognition.

## CONFLICT OF INTEREST

The authors declare that they have no competing interests.

## AUTHORS' CONTRIBUTIONS

Haitao Jiang contributed to data analysis, interpretation, and drafting. Lianhe Zhao contributed to data collection. Yunjie Chen and Liang Sun involved in study design, study supervision, and final approval of the article. All authors read and approved the final article.

## Supporting information

TblS1

## Data Availability

LncRNA transcriptome expression data of patients were downloaded from the Tanric database (https://www.tanric.org/home).
