# Supplementary material for: A four‐long noncoding RNA signature predicts survival of hepatocellular carcinoma patients
Source: J Clin Lab Anal. 2020 May 30;34(9):e23377. doi: 10.1002/jcla.23377 (PMC7521318; doi:10.1002/jcla.23377)
Supplement: Supplementary file 1 — TblS1 [file JCLA-34-e23377-s001.doc]

| **Supplementary Table S1: Genes co-expression genes with the four lncRNAs in the entire expression dataset (Pearson correlation coefficient >0.2, P<0.05, n=180).** | | | |
| --- | --- | --- | --- |
| **molecular markers** | **Co-expression genes** | **Pearson Coefficient** | ***P* Value** |
| ENSG00000249926 | GRIN1 | 0.258797836 | 0.000215385 |
| ENSG00000249926 | SDK2 | 0.32400742 | 2.86E-06 |
| ENSG00000249926 | CHST8 | 0.377967167 | 3.43E-08 |
| ENSG00000249926 | HRH3 | 0.370918281 | 6.42E-08 |
| ENSG00000249926 | CPEB1 | 0.326481864 | 2.38E-06 |
| ENSG00000249926 | BEGAIN | 0.309902515 | 7.98E-06 |
| ENSG00000249926 | IGFBPL1 | 0.231948206 | 0.000950204 |
| ENSG00000249926 | ITGA2B | 0.251492934 | 0.000327817 |
| ENSG00000249926 | AIRE | 0.254338913 | 0.000278738 |
| ENSG00000249926 | DNAJA4 | 0.284649189 | 4.40E-05 |
| ENSG00000249926 | PSD | 0.236405165 | 0.000751088 |
| ENSG00000249926 | LRRC26 | 0.302812684 | 1.31E-05 |
| ENSG00000249926 | BMP3 | 0.237157749 | 0.000721535 |
| ENSG00000249926 | PRDM16 | 0.257918653 | 0.000226701 |
| ENSG00000249926 | PDILT | 0.280969872 | 5.58E-05 |
| ENSG00000249926 | C10orf93 | 0.295598079 | 2.14E-05 |
| ENSG00000249926 | GRIN2D | 0.304544412 | 1.16E-05 |
| ENSG00000249926 | SLC18A2 | 0.290646098 | 2.98E-05 |
| ENSG00000249926 | NRN1 | 0.311238477 | 7.26E-06 |
| ENSG00000249926 | TMEM179 | 0.358353253 | 1.89E-07 |
| ENSG00000249926 | TRIM49 | 0.316503119 | 4.97E-06 |
| ENSG00000249926 | GPR123 | 0.392633891 | 8.90E-09 |
| ENSG00000249926 | SYT2 | 0.389490592 | 1.20E-08 |
| ENSG00000249926 | SYT5 | 0.414520146 | 1.05E-09 |
| ENSG00000249926 | CFD | 0.237821686 | 0.000696356 |
| ENSG00000249926 | TFAP2C | 0.24643694 | 0.000435289 |
| ENSG00000249926 | TFAP2E | 0.23256449 | 0.000920046 |
| ENSG00000249926 | FAM132A | 0.291776475 | 2.76E-05 |
| ENSG00000249926 | OR52J3 | 0.256863315 | 0.000241015 |
| ENSG00000249926 | GNRH2 | 0.2542517 | 0.000280135 |
| ENSG00000249926 | ZFR2 | 0.240670426 | 0.000597255 |
| ENSG00000249926 | DPYSL4 | 0.233491055 | 0.000876356 |
| ENSG00000249926 | MLL4 | 0.27691358 | 7.20E-05 |
| ENSG00000249926 | EPHX3 | 0.317963403 | 4.47E-06 |
| ENSG00000249926 | OR5H15 | 0.253779232 | 0.000287814 |
| ENSG00000249926 | NGB | 0.254972287 | 0.000268789 |
| ENSG00000249926 | EID2B | -0.240023702 | 0.000618536 |
| ENSG00000249926 | NTF3 | 0.242253313 | 0.000547991 |
| ENSG00000249926 | KRT32 | 0.299546882 | 1.64E-05 |
| ENSG00000249926 | NCRNA00112 | 0.254856537 | 0.000270582 |
| ENSG00000249926 | LOC360030 | 0.236501733 | 0.000747234 |
| ENSG00000249926 | KIAA0895 | -0.274056037 | 8.61E-05 |
| ENSG00000249926 | DIO3OS | 0.259805929 | 0.000203058 |
| ENSG00000249926 | SLC7A4 | 0.338355542 | 9.56E-07 |
| ENSG00000249926 | CRABP1 | 0.410960662 | 1.50E-09 |
| ENSG00000249926 | LASS1 | 0.240127327 | 0.00061508 |
| ENSG00000249926 | LOC25845 | 0.332606234 | 1.49E-06 |
| ENSG00000249926 | PPY2 | 0.348467769 | 4.27E-07 |
| ENSG00000249926 | TWISTNB | -0.316674462 | 4.91E-06 |
| ENSG00000249926 | HAR1B | 0.270718442 | 0.00010567 |
| ENSG00000249926 | HAR1A | 0.253101511 | 0.000299171 |
| ENSG00000249926 | CLDN5 | 0.27479957 | 8.22E-05 |
| ENSG00000249926 | RASGEF1A | 0.286970134 | 3.79E-05 |
| ENSG00000249926 | NPTXR | 0.331721725 | 1.60E-06 |
| ENSG00000249926 | PYDC1 | 0.349059128 | 4.07E-07 |
| ENSG00000249926 | CRH | 0.236457218 | 0.000749008 |
| ENSG00000249926 | TMEM121 | 0.256957861 | 0.000239699 |
| ENSG00000249926 | NKX6-2 | 0.594701075 | 1.62E-20 |
| ENSG00000249926 | OXCT2 | 0.256547236 | 0.000245463 |
| ENSG00000249926 | PRRG3 | 0.268862095 | 0.000118322 |
| ENSG00000249926 | MAG | 0.231412557 | 0.000977153 |
| ENSG00000249926 | LEUTX | 0.26646258 | 0.000136782 |
| ENSG00000249926 | VAPA | 0.284162295 | 4.55E-05 |
| ENSG00000249926 | PPP2R2C | 0.250946631 | 0.000338111 |
| ENSG00000249926 | CALY | 0.391208416 | 1.02E-08 |
| ENSG00000249926 | C5orf47 | 0.238989536 | 0.000654026 |
| ENSG00000249926 | KCNG1 | 0.256207594 | 0.000250328 |
| ENSG00000249926 | OR8D4 | 0.289327789 | 3.25E-05 |
| ENSG00000249926 | CKB | 0.251632743 | 0.000325229 |
| ENSG00000249926 | NTSR1 | 0.344186697 | 6.03E-07 |
| ENSG00000249926 | ARHGAP8 | 0.27792211 | 6.76E-05 |
| ENSG00000249926 | LOC100133669 | 0.2951435 | 2.21E-05 |
| ENSG00000249926 | GAL | 0.307370834 | 9.54E-06 |
| ENSG00000249926 | PTP4A3 | 0.255613471 | 0.000259054 |
| ENSG00000249926 | TNNI3 | 0.354598202 | 2.58E-07 |
| ENSG00000249926 | HOXC6 | 0.239340213 | 0.000641786 |
| ENSG00000249926 | MAPK12 | 0.257929307 | 0.00022656 |
| ENSG00000249926 | CKMT1A | 0.260617301 | 0.000193618 |
| ENSG00000249926 | CKMT1B | 0.23585936 | 0.000773215 |
| ENSG00000249926 | ISYNA1 | 0.231505235 | 0.00097244 |
| ENSG00000249926 | C21orf54 | 0.25497855 | 0.000268692 |
| ENSG00000249926 | LOC388428 | 0.396700037 | 6.05E-09 |
| ENSG00000249926 | PRRX2 | 0.302677408 | 1.32E-05 |
| ENSG00000249926 | SRRM3 | 0.282226877 | 5.15E-05 |
| ENSG00000249926 | FAM171A2 | 0.254460139 | 0.000276808 |
| ENSG00000249926 | MYH1 | 0.235013459 | 0.000808696 |
| ENSG00000249926 | OTOP3 | 0.303361462 | 1.26E-05 |
| ENSG00000249926 | BRSK2 | 0.348390469 | 4.30E-07 |
| ENSG00000249926 | BRSK1 | 0.236263946 | 0.000756756 |
| ENSG00000249926 | JPH1 | 0.260462398 | 0.000195388 |
| ENSG00000249926 | JPH4 | 0.2806519 | 5.69E-05 |
| ENSG00000249926 | SLC39A4 | 0.245725894 | 0.000452787 |
| ENSG00000249926 | MYH13 | 0.317388447 | 4.66E-06 |
| ENSG00000249926 | TMEM63C | 0.30237574 | 1.35E-05 |
| ENSG00000249926 | NTN5 | -0.234075431 | 0.000849792 |
| ENSG00000249926 | B4GALNT2 | 0.295260535 | 2.19E-05 |
| ENSG00000249926 | MAGED4B | 0.234179151 | 0.000845155 |
| ENSG00000249926 | GH2 | 0.441710796 | 5.85E-11 |
| ENSG00000249926 | MYH8 | 0.287250456 | 3.72E-05 |
| ENSG00000249926 | KRTAP5-5 | 0.252716791 | 0.000305802 |
| ENSG00000249926 | HHIPL1 | 0.232738051 | 0.000911712 |
| ENSG00000249926 | SLC9A3 | 0.417202363 | 7.96E-10 |
| ENSG00000249926 | SLC18A1 | 0.2969535 | 1.96E-05 |
| ENSG00000249926 | PCBP3 | 0.375847687 | 4.15E-08 |
| ENSG00000249926 | DIRAS1 | 0.286476457 | 3.91E-05 |
| ENSG00000249926 | TOX | 0.233732503 | 0.000865289 |
| ENSG00000249926 | PRNT | 0.464509851 | 4.25E-12 |
| ENSG00000249926 | ATP1B2 | 0.244236647 | 0.000491566 |
| ENSG00000249926 | ZNF343 | -0.25271984 | 0.000305749 |
| ENSG00000249926 | OPRL1 | 0.286530823 | 3.90E-05 |
| ENSG00000249926 | BAI1 | 0.41262476 | 1.27E-09 |
| ENSG00000249926 | TLX2 | 0.312922691 | 6.44E-06 |
| ENSG00000249926 | TLX3 | 0.335535783 | 1.19E-06 |
| ENSG00000249926 | CCKBR | 0.292838364 | 2.58E-05 |
| ENSG00000249926 | C19orf77 | 0.26757314 | 0.000127927 |
| ENSG00000249926 | ESRRB | 0.232001022 | 0.000947585 |
| ENSG00000249926 | FAM19A5 | 0.29258756 | 2.62E-05 |
| ENSG00000249926 | TBX1 | 0.383649237 | 2.05E-08 |
| ENSG00000249926 | CPAMD8 | 0.258707408 | 0.000216524 |
| ENSG00000249926 | LEPREL2 | 0.261466618 | 0.000184176 |
| ENSG00000249926 | LPHN1 | 0.275749602 | 7.75E-05 |
| ENSG00000249926 | DUOXA1 | 0.267166305 | 0.000131107 |
| ENSG00000249926 | GRAPL | 0.30656698 | 1.01E-05 |
| ENSG00000249926 | CNTFR | 0.232737081 | 0.000911759 |
| ENSG00000249926 | KCNH2 | 0.298500385 | 1.76E-05 |
| ENSG00000249926 | RADIL | 0.232358953 | 0.000930005 |
| ENSG00000249926 | KCNF1 | 0.269273372 | 0.000115403 |
| ENSG00000249926 | FLJ42875 | 0.258733727 | 0.000216192 |
| ENSG00000249926 | SLC16A3 | 0.255017091 | 0.000268098 |
| ENSG00000249926 | CACNA1B | 0.42682183 | 2.93E-10 |
| ENSG00000249926 | SLC22A17 | 0.251149351 | 0.000334256 |
| ENSG00000249926 | PPP2R1B | 0.24068997 | 0.000596623 |
| ENSG00000249926 | C11orf20 | 0.246207459 | 0.000440867 |
| ENSG00000249926 | KLK13 | 0.3167452 | 4.89E-06 |
| ENSG00000249926 | UAP1L1 | 0.269664353 | 0.00011269 |
| ENSG00000249926 | SNCG | 0.283583226 | 4.72E-05 |
| ENSG00000249926 | RENBP | 0.242895536 | 0.000529096 |
| ENSG00000249926 | LHB | 0.283717623 | 4.68E-05 |
| ENSG00000249926 | TSPYL1 | -0.242300702 | 0.000546575 |
| ENSG00000249926 | C15orf59 | 0.305325085 | 1.10E-05 |
| ENSG00000249926 | NME4 | 0.264417146 | 0.000154609 |
| ENSG00000249926 | RUNDC3A | 0.325163385 | 2.63E-06 |
| ENSG00000249926 | PCSK1N | 0.243215444 | 0.00051991 |
| ENSG00000249926 | LCN10 | 0.268949503 | 0.000117696 |
| ENSG00000249926 | IQCJ | 0.462381259 | 5.47E-12 |
| ENSG00000249926 | MUC12 | 0.318635638 | 4.26E-06 |
| ENSG00000249926 | C1orf61 | 0.23115449 | 0.000990385 |
| ENSG00000249926 | SLC22A8 | 0.326594788 | 2.36E-06 |
| ENSG00000249926 | UBE2E3 | -0.261618534 | 0.000182533 |
| ENSG00000249926 | GNB5 | 0.231374126 | 0.000979113 |
| ENSG00000249926 | ECEL1 | 0.499995753 | 4.78E-14 |
| ENSG00000249926 | DRD4 | 0.271028813 | 0.000103683 |
| ENSG00000249926 | ODF1 | 0.246424909 | 0.00043558 |
| ENSG00000249926 | EFEMP2 | 0.240994112 | 0.00058686 |
| ENSG00000249926 | SATB2 | -0.306048774 | 1.05E-05 |
| ENSG00000249926 | GATA5 | 0.44502707 | 4.04E-11 |
| ENSG00000249926 | TRIM49L | 0.401835987 | 3.69E-09 |
| ENSG00000249926 | KIF1A | 0.234726637 | 0.000821064 |
| ENSG00000249926 | AATK | 0.29018384 | 3.07E-05 |
| ENSG00000249926 | TRIM74 | 0.258479254 | 0.000219423 |
| ENSG00000249926 | RASAL1 | 0.256346674 | 0.000248325 |
| ENSG00000249926 | ICAM5 | 0.234974951 | 0.000810347 |
| ENSG00000249926 | ICAM4 | 0.277108744 | 7.11E-05 |
| ENSG00000249926 | LY6E | 0.256099513 | 0.000251895 |
| ENSG00000249926 | CDH15 | 0.268457997 | 0.000121258 |
| ENSG00000249926 | C8orf73 | 0.264163232 | 0.000156968 |
| ENSG00000249926 | MIOX | 0.231656923 | 0.000964773 |
| ENSG00000249926 | MMP17 | 0.340950753 | 7.80E-07 |
| ENSG00000249926 | IGLON5 | 0.293626563 | 2.44E-05 |
| ENSG00000249926 | LHFPL4 | 0.240188784 | 0.000613038 |
| ENSG00000249926 | TRIM50 | 0.237933628 | 0.000692192 |
| ENSG00000249926 | WNK4 | 0.243183994 | 0.000520807 |
| ENSG00000249926 | STK32C | 0.277482341 | 6.95E-05 |
| ENSG00000249926 | EGFL7 | 0.244276522 | 0.000490489 |
| ENSG00000249926 | C20orf166 | 0.240310046 | 0.000609029 |
| ENSG00000249926 | KREMEN2 | 0.24409092 | 0.000495522 |
| ENSG00000249926 | PTGES | 0.246077121 | 0.000444064 |
| ENSG00000249926 | C20orf201 | 0.381422665 | 2.51E-08 |
| ENSG00000249926 | PHF21B | 0.322989885 | 3.09E-06 |
| ENSG00000249926 | SLC11A2 | -0.260718815 | 0.000192466 |
| ENSG00000249926 | LOC100270710 | 0.232437376 | 0.000926193 |
| ENSG00000249926 | SLC46A2 | 0.271159237 | 0.000102858 |
| ENSG00000249926 | NAT8L | 0.323832373 | 2.90E-06 |
| ENSG00000249926 | OR4X1 | 0.253216509 | 0.000297215 |
| ENSG00000249926 | PRAMEF12 | 0.28557901 | 4.15E-05 |
| ENSG00000249926 | PRSS21 | 0.250595336 | 0.000344888 |
| ENSG00000249926 | DUOX1 | 0.300544586 | 1.53E-05 |
| ENSG00000249926 | DUOX2 | 0.236480449 | 0.000748082 |
| ENSG00000249926 | IFITM5 | 0.232673496 | 0.000914804 |
| ENSG00000249926 | FAM27B | 0.257374979 | 0.000233973 |
| ENSG00000249926 | ACCN1 | 0.277529074 | 6.93E-05 |
| ENSG00000249926 | BCAN | 0.237262421 | 0.00071751 |
| ENSG00000249926 | TCERG1L | 0.319899053 | 3.88E-06 |
| ENSG00000249926 | KCNQ2 | 0.269114372 | 0.000116523 |
| ENSG00000249926 | PDE6B | 0.420963874 | 5.41E-10 |
| ENSG00000249926 | PTGER1 | 0.242854612 | 0.000530282 |
| ENSG00000249926 | NEFH | 0.24501586 | 0.000470908 |
| ENSG00000249926 | ESYT3 | 0.297320371 | 1.91E-05 |
| ENSG00000249926 | CHRND | 0.2650825 | 0.000148585 |
| ENSG00000249926 | ARHGEF4 | 0.315080019 | 5.51E-06 |
| ENSG00000249926 | PALM | 0.289786349 | 3.15E-05 |
| ENSG00000249926 | OR2V2 | 0.278496425 | 6.52E-05 |
| ENSG00000249926 | RPP25 | 0.287295916 | 3.71E-05 |
| ENSG00000249926 | CILP2 | 0.2362468 | 0.000757447 |
| ENSG00000249926 | MYH4 | 0.264471571 | 0.000154108 |
| ENSG00000249926 | FGD5 | 0.277819621 | 6.80E-05 |
| ENSG00000249926 | PTGDS | 0.324239868 | 2.82E-06 |
| ENSG00000249926 | GLCCI1 | -0.23428763 | 0.00084033 |
| ENSG00000249926 | SLC6A3 | 0.436973095 | 9.85E-11 |
| ENSG00000249926 | POM121L1P | 0.250556332 | 0.000345648 |
| ENSG00000249926 | GSC | 0.235294577 | 0.000796742 |
| ENSG00000249926 | RNF212 | 0.29469618 | 2.28E-05 |
| ENSG00000249926 | RIMS4 | 0.274662183 | 8.29E-05 |
| ENSG00000249926 | TAS2R7 | 0.348520827 | 4.25E-07 |
| ENSG00000249926 | GRAMD4 | 0.231460559 | 0.000974709 |
| ENSG00000249926 | GPR172B | 0.232502541 | 0.000923037 |
| ENSG00000249926 | NMUR1 | 0.256690346 | 0.00024344 |
| ENSG00000249926 | ZNF266 | -0.298133653 | 1.81E-05 |
| ENSG00000249926 | PRSS16 | 0.277379495 | 6.99E-05 |
| ENSG00000249926 | SOX1 | 0.466963833 | 3.17E-12 |
| ENSG00000249926 | LOC100131691 | -0.23847095 | 0.000672521 |
| ENSG00000249926 | FES | 0.247663373 | 0.000406573 |
| ENSG00000249926 | PNMAL2 | 0.250156906 | 0.000353523 |
| ENSG00000249926 | PNMAL1 | 0.327391046 | 2.22E-06 |
| ENSG00000249926 | TTYH2 | 0.236708943 | 0.000739027 |
| ENSG00000249926 | LCNL1 | 0.252735099 | 0.000305484 |
| ENSG00000249926 | LOC254559 | 0.271152808 | 0.000102898 |
| ENSG00000249926 | RYR1 | 0.303337718 | 1.26E-05 |
| ENSG00000249926 | GIPC3 | 0.313048008 | 6.38E-06 |
| ENSG00000249926 | HMX1 | 0.340753045 | 7.92E-07 |
| ENSG00000249926 | SLC7A10 | 0.249850421 | 0.000359679 |
| ENSG00000259681 | C3orf77 | 0.245512387 | 0.000458167 |
| ENSG00000259681 | ZCCHC13 | 0.469578376 | 2.31E-12 |
| ENSG00000259681 | FLJ46361 | 0.312933837 | 6.43E-06 |
| ENSG00000259681 | TMEM84 | 0.299682508 | 1.62E-05 |
| ENSG00000259681 | 11-Mar | 0.244868975 | 0.00047474 |
| ENSG00000259681 | OR8S1 | 0.41825776 | 7.15E-10 |
| ENSG00000259681 | MYF5 | 0.324384976 | 2.78E-06 |
| ENSG00000259681 | SYCP2 | 0.250671045 | 0.000343417 |
| ENSG00000259681 | OR2G3 | 0.458076063 | 9.09E-12 |
| ENSG00000259681 | CNTNAP5 | 0.263031501 | 0.000167894 |
| ENSG00000259681 | OR6Q1 | 0.53446497 | 3.60E-16 |
| ENSG00000259681 | OR2A5 | 0.441387949 | 6.07E-11 |
| ENSG00000259681 | LOC100287718 | 0.243854623 | 0.000501998 |
| ENSG00000259681 | OR8H1 | 0.458076063 | 9.09E-12 |
| ENSG00000259681 | OR6C75 | 0.313660014 | 6.11E-06 |
| ENSG00000259681 | KRTAP24-1 | 0.458076063 | 9.09E-12 |
| ENSG00000259681 | LRIT1 | 0.289987077 | 3.11E-05 |
| ENSG00000259681 | ATXN3L | 0.27890421 | 6.35E-05 |
| ENSG00000259681 | OR4C13 | 0.288056355 | 3.53E-05 |
| ENSG00000259681 | OR4C12 | 0.289157861 | 3.29E-05 |
| ENSG00000259681 | DEFB112 | 0.240784841 | 0.000593562 |
| ENSG00000259681 | ATXN8OS | 0.324097921 | 2.85E-06 |
| ENSG00000259681 | MOG | 0.243730077 | 0.000505443 |
| ENSG00000259681 | ANKRD30A | 0.235284615 | 0.000797163 |
| ENSG00000259681 | SFRS12IP1 | 0.241074984 | 0.000584289 |
| ENSG00000259681 | OR10W1 | 0.338848374 | 9.20E-07 |
| ENSG00000259681 | ATP12A | 0.286340292 | 3.95E-05 |
| ENSG00000259681 | OR2AT4 | 0.292222865 | 2.68E-05 |
| ENSG00000259681 | REXO1L1 | 0.23649639 | 0.000747447 |
| ENSG00000259681 | NOBOX | 0.247400705 | 0.000412571 |
| ENSG00000259681 | DAPL1 | 0.246924213 | 0.000423662 |
| ENSG00000259681 | RGR | 0.247715027 | 0.000405403 |
| ENSG00000259681 | OR51F1 | 0.458076063 | 9.09E-12 |
| ENSG00000259681 | LOC283332 | 0.53446497 | 3.60E-16 |
| ENSG00000259681 | SP8 | 0.234802464 | 0.000817777 |
| ENSG00000259681 | TSPYL6 | 0.348334796 | 4.31E-07 |
| ENSG00000259681 | OR4A15 | 0.308616635 | 8.74E-06 |
| ENSG00000259681 | POTED | 0.293974485 | 2.39E-05 |
| ENSG00000259681 | OR52E8 | 0.346396493 | 5.05E-07 |
| ENSG00000259681 | OR5AP2 | 0.32870878 | 2.01E-06 |
| ENSG00000259681 | OR5D18 | 0.458076063 | 9.09E-12 |
| ENSG00000259681 | FLJ25328 | 0.260049231 | 0.000200183 |
| ENSG00000259681 | LOC150185 | 0.33889436 | 9.17E-07 |
| ENSG00000259681 | GPR148 | 0.241663239 | 0.000565899 |
| ENSG00000259681 | IMP5 | 0.548123874 | 4.44E-17 |
| ENSG00000259681 | GJD2 | 0.233582196 | 0.000872163 |
| ENSG00000259681 | C18orf62 | 0.252970602 | 0.000301413 |
| ENSG00000259681 | OR4A5 | 0.53446497 | 3.60E-16 |
| ENSG00000259681 | LOC730811 | 0.334369018 | 1.30E-06 |
| ENSG00000259681 | KRTAP9-8 | 0.283426217 | 4.77E-05 |
| ENSG00000259681 | OR10G4 | 0.353317291 | 2.87E-07 |
| ENSG00000259681 | LRRC30 | 0.481165702 | 5.51E-13 |
| ENSG00000259681 | FAM71B | 0.332160453 | 1.55E-06 |
| ENSG00000259681 | ZNF705A | 0.233003026 | 0.000899124 |
| ENSG00000259681 | OR10G8 | 0.458076063 | 9.09E-12 |
| ENSG00000259681 | FLJ44082 | 0.513298703 | 7.73E-15 |
| ENSG00000259681 | FLG2 | 0.243048656 | 0.000524681 |
| ENSG00000259681 | COX8C | 0.300251809 | 1.56E-05 |
| ENSG00000259681 | CCDC60 | 0.296120907 | 2.07E-05 |
| ENSG00000259681 | GAST | 0.270504759 | 0.000107059 |
| ENSG00000259681 | OR1L3 | 0.26669186 | 0.000134908 |
| ENSG00000259681 | OR5W2 | 0.458076063 | 9.09E-12 |
| ENSG00000259681 | OR6C65 | 0.458076063 | 9.09E-12 |
| ENSG00000259681 | MSGN1 | 0.332160453 | 1.55E-06 |
| ENSG00000259681 | RMST | 0.235742501 | 0.000778029 |
| ENSG00000259681 | OR4M1 | 0.431950457 | 1.70E-10 |
| ENSG00000259681 | ZNF705D | 0.277122312 | 7.11E-05 |
| ENSG00000259681 | OR5K3 | 0.274782641 | 8.23E-05 |
| ENSG00000259681 | OR4K17 | 0.287016023 | 3.78E-05 |
| ENSG00000259681 | OR52E6 | 0.258398571 | 0.000220457 |
| ENSG00000259681 | TPTE2P3 | 0.231391919 | 0.000978205 |
| ENSG00000259681 | PLUNC | 0.32689393 | 2.31E-06 |
| ENSG00000259681 | MRGPRX1 | 0.231391087 | 0.000978247 |
| ENSG00000259681 | HOXD1 | 0.236365688 | 0.000752669 |
| ENSG00000259681 | NANOS2 | 0.252942318 | 0.000301899 |
| ENSG00000259681 | C10orf40 | 0.513298703 | 7.73E-15 |
| ENSG00000259681 | OR2AK2 | 0.334369018 | 1.30E-06 |
| ENSG00000259681 | LOC727924 | 0.327132789 | 2.27E-06 |
| ENSG00000259681 | OR4Q3 | 0.269074152 | 0.000116809 |
| ENSG00000259681 | RSPH6A | 0.23319499 | 0.000890104 |
| ENSG00000259681 | ADAM7 | 0.262227237 | 0.000176087 |
| ENSG00000259681 | OR52A5 | 0.240784841 | 0.000593562 |
| ENSG00000259681 | FTHL17 | 0.279487906 | 6.12E-05 |
| ENSG00000259681 | TMEM174 | 0.335812719 | 1.17E-06 |
| ENSG00000259681 | LELP1 | 0.328761064 | 2.00E-06 |
| ENSG00000259681 | HDGFL1 | 0.280910256 | 5.60E-05 |
| ENSG00000259681 | OR4X1 | 0.290374855 | 3.03E-05 |
| ENSG00000259681 | OTUD6A | 0.253022346 | 0.000300525 |
| ENSG00000259681 | KRTAP27-1 | 0.332160453 | 1.55E-06 |
| ENSG00000259681 | KRTAP4-2 | 0.23153867 | 0.000970746 |
| ENSG00000259681 | KRTAP4-4 | 0.285605971 | 4.14E-05 |
| ENSG00000259681 | NF1P1 | 0.294711602 | 2.27E-05 |
| ENSG00000259681 | OR2T8 | 0.275724967 | 7.76E-05 |
| ENSG00000259681 | NOX3 | 0.253619129 | 0.00029046 |
| ENSG00000259681 | FGF4 | 0.410969866 | 1.50E-09 |
| ENSG00000259681 | MAGEB10 | 0.238082388 | 0.000686693 |
| ENSG00000259681 | FKSG73 | 0.53446497 | 3.60E-16 |
| ENSG00000259681 | OR2L3 | 0.233841554 | 0.000860333 |
| ENSG00000259681 | C21orf128 | 0.231201748 | 0.00098795 |
| ENSG00000259681 | KIF2B | 0.264479771 | 0.000154032 |
| ENSG00000259681 | GPX6 | 0.392491245 | 9.02E-09 |
| ENSG00000259681 | POTEC | 0.259642994 | 0.000205005 |
| ENSG00000259681 | ANKRD26P1 | 0.285152175 | 4.26E-05 |
| ENSG00000259681 | CSTT | 0.391724791 | 9.69E-09 |
| ENSG00000259681 | OR4C46 | 0.53446497 | 3.60E-16 |
| ENSG00000259681 | IFNA2 | 0.289838228 | 3.14E-05 |
| ENSG00000259681 | C8orf86 | 0.258930699 | 0.000213721 |
| ENSG00000259788 | RBM14 | 0.236507835 | 0.000746991 |
| ENSG00000259788 | DHX9 | 0.230969541 | 0.000999969 |
| ENSG00000259788 | GPSM2 | 0.237347521 | 0.000714254 |
| ENSG00000259788 | ZNF45 | 0.298588218 | 1.75E-05 |
| ENSG00000259788 | ZNF780B | 0.292007515 | 2.72E-05 |
| ENSG00000259788 | OR5P3 | 0.262119324 | 0.000177214 |
| ENSG00000259788 | AAA1 | 0.234121667 | 0.000847722 |
| ENSG00000259788 | OR2C3 | 0.256051902 | 0.000252588 |
| ENSG00000259788 | SKP2 | 0.251268 | 0.000332019 |
| ENSG00000259788 | FANCF | 0.289396092 | 3.23E-05 |
| ENSG00000259788 | FIGNL1 | 0.258347674 | 0.000221111 |
| ENSG00000259788 | BRD8 | 0.249683867 | 0.000363065 |
| ENSG00000259788 | ZNF589 | 0.259439593 | 0.000207459 |
| ENSG00000259788 | OR52N2 | 0.264706138 | 0.000151965 |
| ENSG00000259788 | KRTAP19-3 | 0.299198756 | 1.68E-05 |
| ENSG00000259788 | KRTAP19-1 | 0.343757491 | 6.24E-07 |
| ENSG00000259788 | ERCC6L | 0.246204549 | 0.000440938 |
| ENSG00000259788 | OCRL | 0.28400925 | 4.59E-05 |
| ENSG00000259788 | ORC2L | 0.233102484 | 0.00089444 |
| ENSG00000259788 | ASF1B | 0.248369032 | 0.000390856 |
| ENSG00000259788 | TAS2R10 | 0.233948399 | 0.000855502 |
| ENSG00000259788 | MACROD2 | 0.28603977 | 4.03E-05 |
| ENSG00000259788 | ZNF833 | 0.2497292 | 0.00036214 |
| ENSG00000259788 | SPZ1 | 0.273643443 | 8.83E-05 |
| ENSG00000259788 | ATP6V0A2 | 0.239978764 | 0.000620041 |
| ENSG00000259788 | BRIP1 | 0.253182101 | 0.000297799 |
| ENSG00000259788 | DOT1L | 0.249388042 | 0.000369153 |
| ENSG00000259788 | PER4 | 0.392688249 | 8.85E-09 |
| ENSG00000259788 | STIL | 0.236675259 | 0.000740356 |
| ENSG00000259788 | ZNF98 | 0.233473327 | 0.000877174 |
| ENSG00000259788 | FST | -0.247521549 | 0.000409801 |
| ENSG00000259788 | CRYGA | 0.236540209 | 0.000745704 |
| ENSG00000259788 | FAM72B | 0.253993708 | 0.000284304 |
| ENSG00000259788 | FAM72A | 0.258959233 | 0.000213365 |
| ENSG00000259788 | LOC144776 | 0.281792444 | 5.29E-05 |
| ENSG00000259788 | RAX2 | 0.354875479 | 2.52E-07 |
| ENSG00000259788 | NCAPG2 | 0.265332724 | 0.000146377 |
| ENSG00000259788 | CSN1S2A | 0.256223989 | 0.000250091 |
| ENSG00000259788 | CLTB | -0.233723537 | 0.000865698 |
| ENSG00000259788 | GPR50 | 0.29772846 | 1.86E-05 |
| ENSG00000259788 | LMX1A | 0.242381531 | 0.000544169 |
| ENSG00000259788 | SHC2 | -0.268609553 | 0.000120149 |
| ENSG00000259788 | ETV3L | 0.281170425 | 5.50E-05 |
| ENSG00000259788 | PRKRIR | 0.251290225 | 0.000331602 |
| ENSG00000259788 | C1orf74 | 0.24890047 | 0.000379394 |
| ENSG00000259788 | LEMD3 | 0.24529404 | 0.00046373 |
| ENSG00000259788 | TPTE | 0.235120335 | 0.000804132 |
| ENSG00000259788 | PAXIP1 | 0.232852971 | 0.000906233 |
| ENSG00000259788 | SAT1 | -0.26636905 | 0.000137553 |
| ENSG00000259788 | SPSB1 | -0.246333706 | 0.00043779 |
| ENSG00000259788 | OR2T33 | 0.290578616 | 2.99E-05 |
| ENSG00000259788 | KIAA0513 | -0.273843721 | 8.72E-05 |
| ENSG00000259788 | E2F7 | 0.274610372 | 8.31E-05 |
| ENSG00000259788 | E2F8 | 0.256551493 | 0.000245403 |
| ENSG00000259788 | IFITM2 | -0.287501937 | 3.66E-05 |
| ENSG00000259788 | GAGE2D | 0.241007839 | 0.000586423 |
| ENSG00000259788 | ZBED5 | 0.272586517 | 9.42E-05 |
| ENSG00000259788 | PDCL | 0.239910114 | 0.000622345 |
| ENSG00000259788 | BRCA1 | 0.245715742 | 0.000453042 |
| ENSG00000259788 | H2BFM | 0.27505866 | 8.09E-05 |
| ENSG00000259788 | XRCC3 | 0.232310184 | 0.000932382 |
| ENSG00000259788 | XRCC2 | 0.265915626 | 0.000141351 |
| ENSG00000259788 | LOC100287718 | 0.263088014 | 0.000167332 |
| ENSG00000259788 | LMNB2 | 0.232571738 | 0.000919696 |
| ENSG00000259788 | LMNB1 | 0.24779724 | 0.000403547 |
| ENSG00000259788 | FRMPD2L1 | 0.277481897 | 6.95E-05 |
| ENSG00000259788 | OTOR | 0.297916133 | 1.83E-05 |
| ENSG00000259788 | TRIM24 | 0.238925929 | 0.000656268 |
| ENSG00000259788 | NUP188 | 0.237012088 | 0.00072717 |
| ENSG00000259788 | BCL9 | 0.252227792 | 0.000314428 |
| ENSG00000259788 | ELP4 | 0.270246006 | 0.000108764 |
| ENSG00000259788 | ZBTB39 | 0.270168074 | 0.000109283 |
| ENSG00000259788 | HTR2C | 0.234010564 | 0.000852703 |
| ENSG00000259788 | GAGE12D | 0.256891579 | 0.000240621 |
| ENSG00000259788 | C7orf71 | 0.270582618 | 0.000106551 |
| ENSG00000259788 | RRAGD | -0.239482962 | 0.000636865 |
| ENSG00000259788 | BRCC3 | 0.238680516 | 0.000664989 |
| ENSG00000259788 | NR4A1 | -0.274147426 | 8.56E-05 |
| ENSG00000259788 | CPSF6 | 0.25043561 | 0.000348011 |
| ENSG00000259788 | SMCHD1 | 0.239732807 | 0.000628335 |
| ENSG00000259788 | NUP107 | 0.29444955 | 2.31E-05 |
| ENSG00000259788 | ALG6 | 0.241417836 | 0.000573505 |
| ENSG00000259788 | ACVR2B | 0.259593757 | 0.000205596 |
| ENSG00000259788 | C13orf34 | 0.286404234 | 3.93E-05 |
| ENSG00000259788 | ZNF384 | 0.249250208 | 0.000372022 |
| ENSG00000259788 | FRS2 | 0.241720748 | 0.00056413 |
| ENSG00000259788 | B4GALT4 | 0.233357187 | 0.000882548 |
| ENSG00000259788 | SGOL2 | 0.23983268 | 0.000624954 |
| ENSG00000259788 | CCDC15 | 0.251314568 | 0.000331145 |
| ENSG00000259788 | FOSL2 | -0.236573405 | 0.000744386 |
| ENSG00000259788 | LOC100240726 | 0.24948403 | 0.000367167 |
| ENSG00000259788 | DCP1B | 0.240125287 | 0.000615148 |
| ENSG00000259788 | DKFZp779M0652 | -0.233764623 | 0.000863827 |
| ENSG00000259788 | LGALS13 | 0.280163749 | 5.87E-05 |
| ENSG00000259788 | LGALS14 | 0.319961233 | 3.86E-06 |
| ENSG00000259788 | OR6C3 | 0.32701711 | 2.29E-06 |
| ENSG00000259788 | SSRP1 | 0.249929679 | 0.000358077 |
| ENSG00000259788 | CIT | 0.234966987 | 0.000810688 |
| ENSG00000259788 | C1orf96 | 0.253252515 | 0.000296605 |
| ENSG00000259788 | KRTAP19-5 | 0.284974115 | 4.31E-05 |
| ENSG00000259788 | VMA21 | 0.255875087 | 0.000255177 |
| ENSG00000259788 | COL23A1 | -0.245764368 | 0.000451824 |
| ENSG00000259788 | PRPF3 | 0.236312241 | 0.000754813 |
| ENSG00000259788 | FAM122B | 0.285186346 | 4.25E-05 |
| ENSG00000259788 | ZBTB12 | 0.240365265 | 0.000607211 |
| ENSG00000259788 | CREM | -0.239436084 | 0.000638477 |
| ENSG00000259788 | SH3RF2 | 0.261042113 | 0.00018884 |
| ENSG00000259788 | ZNF221 | 0.243339676 | 0.000516383 |
| ENSG00000259788 | GOLGA6L6 | 0.27565426 | 7.79E-05 |
| ENSG00000259788 | GOLGA6L1 | 0.238379769 | 0.000675822 |
| ENSG00000259788 | ANXA11 | -0.249864995 | 0.000359384 |
| ENSG00000259788 | KMO | -0.273270813 | 9.03E-05 |
| ENSG00000259788 | G2E3 | 0.251299827 | 0.000331422 |
| ENSG00000259788 | ZNF605 | 0.255143363 | 0.000266159 |
| ENSG00000259788 | WDR5B | 0.266484384 | 0.000136603 |
| ENSG00000259788 | SUZ12 | 0.244269943 | 0.000490667 |
| ENSG00000259788 | HTRA1 | -0.232422592 | 0.000926911 |
| ENSG00000259788 | GOLGA8F | 0.323808977 | 2.91E-06 |
| ENSG00000259788 | FAM47A | 0.31040126 | 7.71E-06 |
| ENSG00000259788 | ZNF599 | 0.253700645 | 0.00028911 |
| ENSG00000259788 | ESCO2 | 0.271591211 | 0.00010017 |
| ENSG00000259788 | ESCO1 | 0.233969094 | 0.00085457 |
| ENSG00000259788 | SMARCC1 | 0.232045521 | 0.000945383 |
| ENSG00000259788 | SENP1 | 0.27048556 | 0.000107185 |
| ENSG00000259788 | NCRNA00200 | 0.239662807 | 0.000630714 |
| ENSG00000259788 | ANKRD32 | 0.268833555 | 0.000118528 |
| ENSG00000259788 | NASP | 0.231618978 | 0.000966686 |
| ENSG00000259788 | NCOA5 | 0.266377619 | 0.000137483 |
| ENSG00000259788 | EMID2 | -0.237468352 | 0.000709653 |
| ENSG00000259788 | MTMR1 | 0.261329115 | 0.000185675 |
| ENSG00000259788 | INCENP | 0.233819681 | 0.000861325 |
| ENSG00000259788 | DEPDC1 | 0.234146879 | 0.000846595 |
| ENSG00000259788 | FANCG | 0.274697571 | 8.27E-05 |
| ENSG00000259788 | CDK2 | 0.232528082 | 0.000921803 |
| ENSG00000259788 | ZNF792 | 0.253383161 | 0.000294402 |
| ENSG00000259788 | CCDC99 | 0.233063662 | 0.000896266 |
| ENSG00000259788 | SDS | -0.262076827 | 0.000177659 |
| ENSG00000259788 | CXorf59 | 0.245536265 | 0.000457563 |
| ENSG00000259788 | HAUS6 | 0.237117979 | 0.000723069 |
| ENSG00000259788 | IPO9 | 0.2367522 | 0.000737324 |
| ENSG00000259788 | C6orf167 | 0.252599094 | 0.000307858 |
| ENSG00000259788 | C5orf23 | -0.232554748 | 0.000920515 |
| ENSG00000259788 | ANLN | 0.234379164 | 0.000836279 |
| ENSG00000259788 | CCDC142 | 0.246006183 | 0.000445813 |
| ENSG00000259788 | OR2W5 | 0.232956424 | 0.000901326 |
| ENSG00000259788 | C12orf68 | -0.235359066 | 0.000794022 |
| ENSG00000259788 | MAGEA9B | 0.251589219 | 0.000326033 |
| ENSG00000259788 | GSTA3 | 0.286263082 | 3.97E-05 |
| ENSG00000259788 | ZNF234 | 0.294052954 | 2.38E-05 |
| ENSG00000259788 | MDH1 | -0.33063523 | 1.74E-06 |
| ENSG00000259788 | NRG1 | -0.234087879 | 0.000849234 |
| ENSG00000259788 | MT1X | -0.282897888 | 4.93E-05 |
| ENSG00000259788 | BLVRB | -0.242383194 | 0.00054412 |
| ENSG00000259788 | TRIM77 | 0.290578616 | 2.99E-05 |
| ENSG00000259788 | ZNF230 | 0.268195119 | 0.000123205 |
| ENSG00000259788 | PDCL2 | 0.231591192 | 0.000968089 |
| ENSG00000259788 | GXYLT1 | 0.263638218 | 0.000161951 |
| ENSG00000259788 | USP37 | 0.245975867 | 0.000446563 |
| ENSG00000259788 | QSER1 | 0.275654387 | 7.79E-05 |
| ENSG00000259788 | SASS6 | 0.258587715 | 0.00021804 |
| ENSG00000259788 | DCP2 | 0.255396766 | 0.000262307 |
| ENSG00000259788 | C12orf48 | 0.236043625 | 0.000765678 |
| ENSG00000259788 | KRTAP10-4 | 0.290140487 | 3.08E-05 |
| ENSG00000259788 | ZMYM3 | 0.239527705 | 0.000635329 |
| ENSG00000259788 | ZMYM1 | 0.278477921 | 6.53E-05 |
| ENSG00000259788 | RASD1 | -0.260709156 | 0.000192576 |
| ENSG00000259788 | RRM1 | 0.293993598 | 2.39E-05 |
| ENSG00000259788 | TXNL1 | -0.356453432 | 2.21E-07 |
| ENSG00000259788 | SART3 | 0.233454767 | 0.000878031 |
| ENSG00000259788 | C1QTNF1 | -0.24501418 | 0.000470952 |
| ENSG00000259788 | OR2L8 | 0.290578616 | 2.99E-05 |
| ENSG00000259788 | C11orf46 | 0.243782498 | 0.00050399 |
| ENSG00000259788 | SUV39H2 | 0.25361023 | 0.000290608 |
| ENSG00000259788 | THOC2 | 0.239313971 | 0.000642695 |
| ENSG00000259788 | TAF4 | 0.256201588 | 0.000250415 |
| ENSG00000259788 | LBR | 0.2451042 | 0.000468618 |
| ENSG00000259788 | ZFP112 | 0.244692347 | 0.000479386 |
| ENSG00000259788 | SLC12A4 | -0.303163267 | 1.28E-05 |
| ENSG00000259788 | RBM12B | 0.269971364 | 0.000110601 |
| ENSG00000259788 | TIMELESS | 0.259423287 | 0.000207657 |
| ENSG00000259788 | C12orf27 | 0.234004803 | 0.000852962 |
| ENSG00000259788 | COMTD1 | -0.2493455 | 0.000370036 |
| ENSG00000259788 | AKR1C1 | -0.32717986 | 2.26E-06 |
| ENSG00000259788 | AKR1C2 | -0.32905744 | 1.96E-06 |
| ENSG00000259788 | ZNF530 | 0.247173371 | 0.000417828 |
| ENSG00000259788 | TCF25 | -0.295672199 | 2.13E-05 |
| ENSG00000259788 | CXorf30 | 0.295976068 | 2.09E-05 |
| ENSG00000259788 | UBAP2L | 0.235291641 | 0.000796866 |
| ENSG00000259788 | TOPBP1 | 0.23106939 | 0.000994784 |
| ENSG00000259788 | GADD45B | -0.285172073 | 4.26E-05 |
| ENSG00000259788 | SPEF2 | 0.288483927 | 3.43E-05 |
| ENSG00000259788 | ZNF273 | 0.234131559 | 0.00084728 |
| ENSG00000259788 | CTXN2 | 0.276282937 | 7.49E-05 |
| ENSG00000259788 | TRPM4 | -0.231275749 | 0.000984147 |
| ENSG00000259788 | ZNF445 | 0.239078765 | 0.000650891 |
| ENSG00000259788 | ZWILCH | 0.255621625 | 0.000258933 |
| ENSG00000259788 | DQX1 | 0.240719435 | 0.000595671 |
| ENSG00000259788 | EED | 0.239920023 | 0.000622012 |
| ENSG00000259788 | ZNF235 | 0.269195206 | 0.000115952 |
| ENSG00000259788 | LOC284632 | 0.392936543 | 8.65E-09 |
| ENSG00000259788 | FKTN | 0.255522705 | 0.000260412 |
| ENSG00000259788 | TTTY5 | 0.28567949 | 4.12E-05 |
| ENSG00000259788 | USP1 | 0.261660247 | 0.000182084 |
| ENSG00000259788 | RGS21 | 0.295042384 | 2.22E-05 |
| ENSG00000259788 | CDCA4 | 0.241252804 | 0.000578673 |
| ENSG00000259788 | CDCA8 | 0.267966333 | 0.000124922 |
| ENSG00000259788 | PLCH1 | 0.235247405 | 0.000798736 |
| ENSG00000259788 | KRTAP6-3 | 0.283512225 | 4.74E-05 |
| ENSG00000259788 | MAGEA10 | 0.233448595 | 0.000878316 |
| ENSG00000259788 | SLC22A17 | -0.246492613 | 0.000433946 |
| ENSG00000259788 | C3orf63 | 0.236120492 | 0.000762555 |
| ENSG00000259788 | CHEK1 | 0.249410585 | 0.000368686 |
| ENSG00000259788 | GINS1 | 0.233018707 | 0.000898384 |
| ENSG00000259788 | CHCHD10 | -0.254234404 | 0.000280413 |
| ENSG00000259788 | PSMA8 | 0.314955184 | 5.56E-06 |
| ENSG00000259788 | ANGEL2 | 0.233990629 | 0.0008536 |
| ENSG00000259788 | TGIF2LX | 0.232058253 | 0.000944753 |
| ENSG00000259788 | CCNF | 0.293403972 | 2.48E-05 |
| ENSG00000259788 | CDC25A | 0.235298854 | 0.000796561 |
| ENSG00000259788 | RBPMS2 | -0.293104035 | 2.53E-05 |
| ENSG00000259788 | MRE11A | 0.286098661 | 4.01E-05 |
| ENSG00000259788 | ZNF84 | 0.26968521 | 0.000112547 |
| ENSG00000259788 | DDX12 | 0.233648926 | 0.000869105 |
| ENSG00000259788 | KIF18A | 0.267615716 | 0.000127598 |
| ENSG00000259788 | BRP44L | -0.234717809 | 0.000821447 |
| ENSG00000259788 | RNASE11 | 0.252006988 | 0.000318397 |
| ENSG00000259788 | OR2AG1 | 0.290578616 | 2.99E-05 |
| ENSG00000259788 | DIDO1 | 0.264412531 | 0.000154652 |
| ENSG00000259788 | MSH2 | 0.298331583 | 1.78E-05 |
| ENSG00000259788 | CLSPN | 0.239480353 | 0.000636954 |
| ENSG00000259788 | MATR3 | 0.256611437 | 0.000244553 |
| ENSG00000259788 | LOC400657 | 0.243581997 | 0.000509567 |
| ENSG00000259788 | SFPQ | 0.238585205 | 0.000668405 |
| ENSG00000259788 | GPR83 | 0.250981813 | 0.000337439 |
| ENSG00000259788 | ANXA6 | -0.234412237 | 0.00083482 |
| ENSG00000259788 | CSH2 | 0.251506676 | 0.000327562 |
| ENSG00000259788 | ATXN7L3B | 0.252199651 | 0.000314931 |
| ENSG00000259788 | CCDC66 | 0.264014916 | 0.000158361 |
| ENSG00000259788 | CDC23 | 0.28298082 | 4.90E-05 |
| ENSG00000259788 | BCOR | 0.241260915 | 0.000578418 |
| ENSG00000259788 | HCFC1 | 0.258965899 | 0.000213282 |
| ENSG00000259788 | HPSE2 | 0.232556367 | 0.000920437 |
| ENSG00000259788 | ZNF687 | 0.244163268 | 0.000493554 |
| ENSG00000259788 | IQCC | 0.258398989 | 0.000220451 |
| ENSG00000259788 | SPIN3 | 0.262418757 | 0.000174103 |
| ENSG00000259788 | NCAPD3 | 0.268841286 | 0.000118472 |
| ENSG00000259788 | GPRC5D | 0.259386532 | 0.000208104 |
| ENSG00000259788 | NEDD1 | 0.242149586 | 0.0005511 |
| ENSG00000259788 | SMC2 | 0.251650852 | 0.000324896 |
| ENSG00000259788 | DDX55 | 0.269771459 | 0.000111957 |
| ENSG00000259788 | RNASEN | 0.27922792 | 6.23E-05 |
| ENSG00000259788 | MCM2 | 0.235168381 | 0.000802088 |
| ENSG00000259788 | CCDC41 | 0.237609046 | 0.000704331 |
| ENSG00000259788 | MCM9 | 0.263293065 | 0.000165308 |
| ENSG00000259788 | C11orf87 | 0.252450921 | 0.000310465 |
| ENSG00000259788 | RBMX | 0.231444201 | 0.000975541 |
| ENSG00000259788 | ILF3 | 0.240141708 | 0.000614602 |
| ENSG00000259788 | ZNF227 | 0.260157617 | 0.000198915 |
| ENSG00000259788 | CCDC163P | 0.260241011 | 0.000197944 |
| ENSG00000259788 | GAGE8 | 0.23567778 | 0.000780708 |
| ENSG00000259788 | TCHP | 0.307441845 | 9.50E-06 |
| ENSG00000259788 | SNORA36A | 0.234713929 | 0.000821616 |
| ENSG00000259788 | GLE1 | 0.264784928 | 0.000151251 |
| ENSG00000259788 | PRELID2 | 0.232850251 | 0.000906362 |
| ENSG00000259788 | TGIF2LY | 0.241651692 | 0.000566255 |
| ENSG00000259788 | TAS2R9 | 0.390763719 | 1.06E-08 |
| ENSG00000259788 | HIC2 | 0.239921565 | 0.00062196 |
| ENSG00000259788 | ASPM | 0.233245872 | 0.000887727 |
| ENSG00000259788 | VEZT | 0.242584285 | 0.000538177 |
| ENSG00000259788 | KNTC1 | 0.255324392 | 0.000263402 |
| ENSG00000259788 | HNRNPH1 | 0.250279207 | 0.000351095 |
| ENSG00000259788 | RBM12 | 0.246324008 | 0.000438026 |
| ENSG00000259788 | PRPF38A | 0.238546746 | 0.000669788 |
| ENSG00000259788 | KIAA0528 | 0.260873695 | 0.000190721 |
| ENSG00000259788 | PASK | 0.247419778 | 0.000412133 |
| ENSG00000259788 | GATA4 | 0.243385288 | 0.000515094 |
| ENSG00000259788 | HMMR | 0.239893347 | 0.000622909 |
| ENSG00000259788 | PRMT3 | 0.302733284 | 1.32E-05 |
| ENSG00000259788 | NUP155 | 0.243584697 | 0.000509492 |
| ENSG00000259788 | CEP192 | 0.247612004 | 0.000407739 |
| ENSG00000259788 | ZFC3H1 | 0.281981015 | 5.23E-05 |
| ENSG00000259788 | IGF2BP3 | 0.243918825 | 0.000500231 |
| ENSG00000259788 | COPB1 | 0.238096029 | 0.000686191 |
| ENSG00000259788 | SOX13 | 0.238296182 | 0.000678862 |
| ENSG00000259788 | TRIM71 | 0.24083482 | 0.000591955 |
| ENSG00000259788 | RPAP3 | 0.235194653 | 0.000800972 |
| ENSG00000259788 | CDH16 | 0.23688853 | 0.000731981 |
| ENSG00000259788 | LOC100128191 | 0.284304462 | 4.50E-05 |
| ENSG00000259788 | SFMBT1 | 0.251334759 | 0.000330767 |
| ENSG00000259788 | CKAP2 | 0.307210988 | 9.65E-06 |
| ENSG00000259788 | CKAP5 | 0.278992558 | 6.32E-05 |
| ENSG00000259788 | CYP39A1 | -0.234455408 | 0.000832919 |
| ENSG00000259788 | C1orf203 | 0.257632849 | 0.000230497 |
| ENSG00000259788 | C5orf54 | 0.309024019 | 8.49E-06 |
| ENSG00000259788 | KIAA0907 | 0.261570563 | 0.00018305 |
| ENSG00000259788 | XPO7 | 0.235067486 | 0.000806386 |
| ENSG00000259788 | ATAD5 | 0.264907648 | 0.000150146 |
| ENSG00000259788 | ESPL1 | 0.271904024 | 9.83E-05 |
| ENSG00000259788 | NFRKB | 0.314974495 | 5.56E-06 |
| ENSG00000259788 | MAGEA5 | 0.239746653 | 0.000627865 |
| ENSG00000259788 | ZNF681 | 0.260483077 | 0.000195151 |
| ENSG00000259788 | WDHD1 | 0.2826435 | 5.01E-05 |
| ENSG00000259788 | ?|317712 | 0.232450854 | 0.00092554 |
| ENSG00000259788 | SR140 | 0.23735318 | 0.000714038 |
| ENSG00000259788 | NCAPD2 | 0.274456961 | 8.39E-05 |
| ENSG00000259788 | POLA1 | 0.269842257 | 0.000111475 |
| ENSG00000259788 | KIAA0556 | 0.246232264 | 0.000440261 |
| ENSG00000259788 | LOC284232 | 0.238790895 | 0.000661054 |
| ENSG00000259788 | JUND | -0.270236261 | 0.000108829 |
| ENSG00000259788 | OR7E156P | 0.248797749 | 0.000381585 |
| ENSG00000259788 | DKC1 | 0.244372944 | 0.000487894 |
| ENSG00000259788 | ZMYM4 | 0.268142851 | 0.000123595 |
| ENSG00000259788 | HOXB1 | 0.270135025 | 0.000109503 |
| ENSG00000259788 | NARG2 | 0.273646793 | 8.83E-05 |
| ENSG00000259788 | RFC5 | 0.277560193 | 6.92E-05 |
| ENSG00000259788 | NUP37 | 0.25135596 | 0.00033037 |
| ENSG00000259788 | OR4F29 | 0.24910565 | 0.000375053 |
| ENSG00000259788 | SEPT14 | 0.237758275 | 0.000698726 |
| ENSG00000259788 | PLAGL2 | 0.285916613 | 4.06E-05 |
| ENSG00000259788 | MCM8 | 0.232807932 | 0.000908377 |
| ENSG00000259788 | EXO1 | 0.242104675 | 0.000552452 |
| ENSG00000259788 | SLC24A2 | 0.233442948 | 0.000878577 |
| ENSG00000259788 | CXorf48 | 0.305832743 | 1.06E-05 |
| ENSG00000259788 | SLITRK1 | 0.262180068 | 0.000176578 |
| ENSG00000259788 | OR8B8 | 0.309335213 | 8.31E-06 |
| ENSG00000259788 | PRAMEF10 | -0.243327717 | 0.000516722 |
| ENSG00000259788 | VENTXP7 | 0.273227165 | 9.06E-05 |
| ENSG00000259788 | DECR1 | -0.26857324 | 0.000120414 |
| ENSG00000259788 | ZNF782 | 0.24999179 | 0.000356827 |
| ENSG00000259788 | GOT1 | -0.236753901 | 0.000737257 |
| ENSG00000259788 | TOP2B | 0.272685867 | 9.37E-05 |
| ENSG00000259788 | HTA | 0.266107161 | 0.000139735 |
| ENSG00000259788 | RBL1 | 0.248770851 | 0.000382161 |
| ENSG00000259788 | MTF2 | 0.251889211 | 0.000320532 |
| ENSG00000259788 | C21orf63 | -0.23115977 | 0.000990113 |
| ENSG00000259788 | IKBKAP | 0.239944267 | 0.000621198 |
| ENSG00000259788 | C12orf74 | 0.276737835 | 7.28E-05 |
| ENSG00000259788 | DGKB | 0.232740141 | 0.000911612 |
| ENSG00000259788 | IQCB1 | 0.259510727 | 0.000206598 |
| ENSG00000259788 | C10orf116 | -0.259872079 | 0.000202273 |
| ENSG00000259788 | CXorf61 | 0.260814776 | 0.000191383 |
| ENSG00000259788 | TADA2A | 0.25825909 | 0.000222255 |
| ENSG00000259788 | ZNF225 | 0.243208495 | 0.000520108 |
| ENSG00000259788 | UBE2E2 | -0.278990338 | 6.32E-05 |
| ENSG00000259788 | PRIM1 | 0.274560111 | 8.34E-05 |
| ENSG00000259788 | QRICH1 | 0.241041978 | 0.000585337 |
| ENSG00000259788 | CENPK | 0.23347019 | 0.000877319 |
| ENSG00000259788 | CENPI | 0.231657928 | 0.000964722 |
| ENSG00000259788 | CENPF | 0.247256432 | 0.0004159 |
| ENSG00000259788 | RAMP1 | -0.2373736 | 0.000713258 |
| ENSG00000259788 | SERPINI2 | 0.274146354 | 8.56E-05 |
| ENSG00000259788 | LIN9 | 0.272000164 | 9.77E-05 |
| ENSG00000259788 | EZH2 | 0.250882241 | 0.000339344 |
| ENSG00000259788 | PNLIPRP2 | 0.266769358 | 0.00013428 |
| ENSG00000259788 | WDR49 | 0.264823284 | 0.000150905 |
| ENSG00000259788 | MAPRE3 | -0.24537842 | 0.000461573 |
| ENSG00000259788 | ANKRD49 | 0.309683776 | 8.11E-06 |
| ENSG00000259788 | NR1H2 | -0.246917256 | 0.000423826 |
| ENSG00000259788 | GK2 | 0.327269628 | 2.24E-06 |
| ENSG00000259788 | ARID3B | 0.293774991 | 2.42E-05 |
| ENSG00000259788 | PUS7L | 0.247439318 | 0.000411684 |
| ENSG00000259788 | DARC | -0.271344439 | 0.000101697 |
| ENSG00000259788 | CAPN7 | 0.233504752 | 0.000875725 |
| ENSG00000259788 | DLEU2 | 0.249795801 | 0.000360786 |
| ENSG00000259788 | LCE6A | 0.314084731 | 5.92E-06 |
| ENSG00000259788 | CLDN18 | 0.271606156 | 0.000100078 |
| ENSG00000259788 | ZNF740 | 0.244352136 | 0.000488453 |
| ENSG00000259788 | TUG1 | 0.23917671 | 0.000647466 |
| ENSG00000259788 | DTL | 0.243326845 | 0.000516747 |
| ENSG00000259788 | C12orf32 | 0.272699597 | 9.36E-05 |
| ENSG00000259788 | RAD51AP1 | 0.236880996 | 0.000732276 |
| ENSG00000259788 | MT2A | -0.271279447 | 0.000102103 |
| ENSG00000259788 | ZNF12 | 0.248268322 | 0.000393064 |
| ENSG00000259788 | KIAA1731 | 0.313138295 | 6.34E-06 |
| ENSG00000259788 | FAM111B | 0.239596132 | 0.000632988 |
| ENSG00000259788 | FAM111A | 0.293449659 | 2.47E-05 |
| ENSG00000259788 | ARID1A | 0.254192802 | 0.000281082 |
| ENSG00000259788 | RAB41 | 0.251662257 | 0.000324686 |
| ENSG00000259788 | AURKAPS1 | 0.233696913 | 0.000866912 |
| ENSG00000259788 | CEBPB | -0.256840115 | 0.000241339 |
| ENSG00000259788 | INTS4 | 0.245211191 | 0.000465857 |
| ENSG00000259788 | OR7E5P | 0.275307633 | 7.96E-05 |
| ENSG00000259788 | KRTAP20-4 | 0.27213412 | 9.69E-05 |
| ENSG00000259788 | LRTM2 | 0.275311874 | 7.96E-05 |
| ENSG00000259788 | FAM13B | 0.232135186 | 0.00094096 |
| ENSG00000259788 | POLE2 | 0.245781075 | 0.000451406 |
| ENSG00000259788 | GGNBP2 | 0.23622217 | 0.000758441 |
| ENSG00000259788 | PHF6 | 0.256160611 | 0.000251008 |
| ENSG00000259788 | C9orf100 | 0.249702418 | 0.000362686 |
| ENSG00000259788 | ZNF283 | 0.237901229 | 0.000693395 |
| ENSG00000259788 | RFXAP | 0.282323917 | 5.11E-05 |
| ENSG00000259788 | CAND1 | 0.241569304 | 0.000568799 |
| ENSG00000259788 | PLEKHG7 | 0.248501348 | 0.000387973 |
| ENSG00000260131 | NKAIN3 | 0.235302255 | 0.000796418 |
| ENSG00000260131 | SLC15A4 | -0.231777507 | 0.000958717 |
| ENSG00000260131 | DPCR1 | 0.231100147 | 0.000993192 |
| ENSG00000260131 | PTH2 | 0.278498961 | 6.52E-05 |
| ENSG00000260131 | VIP | -0.232709955 | 0.000913057 |
| ENSG00000260131 | GLT6D1 | 0.280993145 | 5.57E-05 |
| ENSG00000260131 | CRYBA4 | 0.232159192 | 0.000939779 |
| ENSG00000260131 | FAM96A | -0.243939615 | 0.00049966 |
| ENSG00000260131 | LOC157627 | 0.261282825 | 0.000186182 |
| ENSG00000260131 | SNORA52 | 0.242236018 | 0.000548508 |
| ENSG00000260131 | AGBL1 | 0.401271729 | 3.89E-09 |
| ENSG00000260131 | IFNK | 0.253710136 | 0.000288953 |
| ENSG00000260131 | ALKBH1 | -0.233553239 | 0.000873493 |
| ENSG00000260131 | SNORA58 | 0.269909055 | 0.000111022 |
| ENSG00000260131 | ZAR1L | 0.232564135 | 0.000920063 |
| ENSG00000260131 | P2RY4 | 0.323961678 | 2.87E-06 |
| ENSG00000260131 | DIRC1 | 0.258978107 | 0.00021313 |
| ENSG00000260131 | IL1F10 | 0.330403243 | 1.77E-06 |
| ENSG00000260131 | RPL22 | -0.238613869 | 0.000667376 |
| ENSG00000260131 | PRDM14 | 0.271298397 | 0.000101985 |
| ENSG00000260131 | MUC21 | 0.4382243 | 8.59E-11 |
